# Estimating Urban Road GPS Environment Friendliness with Bus Trajectories: A City-Scale Approach †

**DOI:** 10.3390/s20061580

**Published:** 2020-03-12

**Authors:** Liantao Ma, Chaohe Zhang, Yasha Wang, Guangju Peng, Chao Chen, Junfeng Zhao, Jiangtao Wang

**Affiliations:** 1 Key Laboratory of High Confidence Software Technologies, Ministry of Education, Beijing 100871, China; malt@pku.edu.cn (L.M.); choc@pku.edu.cn (C.Z.); pgj.pku12@pku.edu.cn (G.P.); zhaojf@pku.edu.cn (J.Z.); 2 School of Electronics Engineering and Computer Science, Peking University, Beijing 100871, China; 3 National Engineering Research Center for Software Engineering, Peking University, Beijing 100871, China; 4 College of Computer Science, Chongqing University, Chongqing 400044, China; cschaochen@cqu.edu.cn; 5 School of Computing and Communications, Lancaster University, Lancaster LA1 4YW, UK; jiangtao.wang@lancaster.ac.uk

**Keywords:** location-based service, GPS positioning error, map matching, matrix completion

## Abstract

GPS is taken as the most prevalent positioning system in practice. However, in urban areas, as the GPS satellite signal could be blocked by buildings, the GPS positioning is not accurate due to multi-path errors. Estimating the negative impact of urban environments on GPS accuracy, that is the GPS environment friendliness (GEF) in this paper, will help to predict the GPS errors in different road segments. It enhances user experiences of location-based services and helps to determine where to deploy auxiliary assistant positioning devices. In this paper, we propose a method of processing and analysing massive historical bus GPS trajectory data to estimate the urban road GEF integrated with the contextual information of roads. First, our approach takes full advantage of the particular feature that bus routes are fixed to improve the performance of map matching. In order to estimate the GEF of all roads fairly and reasonably, the method estimates the GPS positioning error of each bus on the roads that are not covered by its route, by taking POIinformation, tag information of roads, and building layout information into account. Finally, we utilize a weighted estimation strategy to calculate the GEF of each road based on the GPS positioning performance of all buses. Based on one month of GPS trajectory data of 4835 buses within the second ring road in Chengdu, China, we estimate the GEF of 8831 different road segments and verify the rationality of the results by satellite maps, street views, and field tests.

## 1. Introduction

GPS is widely used in many location-based services (LBS), such as traffic, tourism, and social interaction. However, the error of GPS positioning has negative impacts on LBS users and even leads to decision-making mistakes. For example, the British police once broke into the home of an innocent person due to a GPS error [1]. Although some methods can improve the accuracy of GPS positioning (e.g., increasing the number of satellites and the vector tracking based on Kalman filtering), they are not effective enough to reduce multipath errors [2,3,4].

Concretely, the multipath effect refers to the phenomenon in urban canyons (e.g., urban areas with tall buildings, overpasses, or street trees); GPS signals cannot reach the receiver through the line of sight (LoS), but are reflected via the building surface or the ground. Recent works [5,6,7,8] confirmed by reassuring experiments that the multipath effect, especially in a built-up urban area, has a major impact on the precision of GPS positioning. Besides, according to the indication generated in the statement of the National Marine Electronics Association (NMEA), the locating information included in the GPS raw data can be used to measure satellite constellations’ geometry errors and receivers’ instrumental errors to some extent. However, the work in [5] indicated that when the primary signal is reflected, the additional distance travelled by the signal due to the reflection can inflate the pseudorange estimate, which cannot be reliably distinguished by GPS receivers. It is not enough to measure the multipath error or GPS positioning error definitely based only on such information [7,9].

To measure the impact that the urban environment has on GPS positioning accuracy, we define GPS environment friendliness (GEF) as the metric: the more negative the effect of the building layout environment on GPS accuracy in a certain area, the poorer the GEF is in this area. The estimation of GEF information in different areas is a fundamental work: First, it helps to improve the user experience of location-based services while the GPS accuracy is limited. For example, if a driver using a ride-sharing app (e.g., Uber) finds that a passenger is located in a poor-GEF area, instead of relying solely on the GPS location information, the driver may choose to communicate the location details with the passenger in advance through a phone call, which reduces the risk of detouring caused by GPS positioning errors. Second, estimating GEF is also helpful for improving the accuracy of GPS in urban areas. There are many methods to improve the accuracy of GPS by combining GPS samples with other complementary information, such as Wi-Fi fingerprints [10], street-view media [11], and 3D-maps [12]. However, the implementation of those solutions often introduces extra costs, such as deploying Wi-Fi access points or updating Wi-Fi fingerprints. GEF can remind people which locations have the most urgent need to deploy an assisted positioning solution (i.e., the locations with the worst GEF) to minimize the overall cost while achieving satisfactory positioning accuracy.

Researchers have conducted several interesting studies on GPS accuracy in different urban areas. For example, Schipperijn [13] selected four routes and recorded 68,000 GPS points to test the dynamic accuracy of the GPS positioning. Drawil [7] proposed a scheme to address localization accuracy estimation utilizing the GPS dataset collected by a vehicle and the knowledge about the surrounding environment. However, most of these have been small-scale and road-by-road field studies. They usually selected some representative streets or locations to evaluate their environmental influence on positioning accuracy. These effort-consuming approaches were only able to estimate the GEF in a limited number of locations. It was difficult to provide a comprehensive city-scale evaluation.

To this end, this paper proposes an approach to estimate the city-scale GEF based on the historical GPS trajectory data of buses. The basic idea is first to divide the urban road network into short and equal-length road segments so that GEF at different locations within the same segment can be treated as the same. Then, we estimate the error of each GPS localization record by using historical bus GPS data and the bus routes’ information. Finally, we statistically analyse the positioning error of the buses on different road segments and calculate their GEF level.

Although the above basic idea is easy to be understand, our proposed approach is not straightforward, since we encounter the following challenges. The routes of all buses in total have a high coverage for roads in a city, while the trajectory data of a single bus can only cover a small part of the road network. It is impossible to assess the GEF of all roads by simply using the GPS data of a single bus. Furthermore, there is a significant variance in the quality of GPS receivers among different buses, which means that the GPS positioning accuracy of different buses on the same road may differ from each other. This may lead to an incorrect conclusion if we estimate the GEF of a road only depending on the buses whose routes cover the road. The GPS samples of different buses cannot be simply aggregated to solve the problem. Therefore, when integrating the GPS data of different buses, we need to develop more sophisticated mechanisms to eliminate the influence brought by the variance of GPS receivers’ quality, so as to compare the GEF on different road segments. The main contributions of the paper are:

- We estimate the GEF of roads at the city scale using the historical GPS trajectories of buses, without the need for extra specialized efforts in GPS data collection. Compared to other methods, this makes our method more scalable, less costly, and more accessible to be transferred to other cities, by only using already existing bus trajectory data. Besides, buses are supposed to run on fixed routes many times a month, which is the prior knowledge for map matching. This helps improve the accuracy and efficiency in the map matching process and reduce the misestimation brought by accidental factors (e.g., the position of satellites, weather).
- We propose a novel three-phase framework for estimating the GEF of urban roads. First, the bus routes’ data and the historical bus GPS data are mapped to the road network based on the map matching algorithm. We calculate the errors of each bus on road segments through which it passes. Secondly, we propose a matrix completion-based method, which makes full use of the correlation between the GPS errors of buses on different road segments and uses the third-party data of urban environment information as regularization to infer the GPS errors of buses on all road segments. Finally, we integrate the errors of buses on all road segments to estimate the GEF.
- We conduct an evaluation and verify our estimated GEF by comparing it with the ground truth collected through field study and the street views on some road segments. The results confirm the effectiveness of our proposed evaluation approach.

## 2. Related Work

### 2.1. GPS Error and Calibration

GPS data have attracted much attention among data mining researchers. Most of the works comprehensively leveraged multiple machine learning techniques, combining GPS data with multi-source heterogeneous data, e.g., POIdata, crowd movement data, etc., to analyse and discover knowledge of a city and further resolve problems in constructing a smart city [14,15,16,17,18,19,20,21,22,23].

However, most works referring to GPS data suffer critical misguidance by GPS positioning error. There are three main components of GPS error [24,25,26,27] including Satellite clock error, signal transmission error (e.g., ionospheric delay, tropospheric delay, multipath effect), and the GPS terminal device’s error. Recent works [7,8] confirmed from reassuring experiments that the multipath effect, especially in built-up urban areas, has a major impact on the precision of GPS positioning. Although many methods can improve the accuracy of GPS positioning, they are not effective enough to reduce multipath errors [2,3,4]. Wu et al. [28] proposed a novel error reduction system for trajectories. However, this approach is designed for sequential localization trajectories and thus cannot figure out the true position of any single GPS positioning record. Wu et al. [29] proposed a model to locate a single GPS position accurately, which was the first work to locate one GPS position as a road. However, the training data of this model relied on the desirable results of map matching, which also encounters problems in urban canyons and tunnels. The possible variance of the quality of GPS receivers was not taken into account.

To obtain a reliable position in urban areas, there are also some existing positioning techniques incorporating GPS data with extra information, such as Wi-Fi fingerprints [10], street view videos/images [11], and 3D maps [12]. In order to maximize the benefits and minimize the total cost, decision-makers should select where to deploy expensive devices cautiously to collect such complementary information. The introduction of GEF provides economic guidance of where to map out those devices.

### 2.2. Measuring GPS Positioning Performance

Researchers have thoroughly studied GPS positioning errors and their causes. GPS receivers cannot reliably distinguish between reflected and direct signals [5]. Besides, there is an indication of the satellite geometry effect on the accuracy, which is called the dilution of precision (DOP) in the GPS measurement data according to the National Marine Electronics Association’s (NMEA) statements [30]. However, the work in [9] indicated that the DOP of the site varies throughout the day. The work in [7] also indicated that although the DOP as a feature shows some power to figure out the positioning performance of a given measurement, it cannot be relied on to perform measurement accuracy classification.

In order to measure the precision of GPS records, data in various scenarios are collected. The work in [31] proposed an urban road friendliness evaluation approach to evaluate GPS positioning accuracy only based on the vehicle trajectory data. The work in [7] proposed a scheme to address localization accuracy estimation by using a vehicle equipped with a standard GPS receiver to collect 6520 real-life GPS measurements. Knowledge about the surrounding environment was also utilized to optimize the classification performance. Modsching [32] gathered positioning data with several facilities at 4000 points in a mid-sized city. The work in [8] selected a few typical zones in the city and then collected GPS data in those places. The work in [13] collected information only from a closely spaced body building apparatus in an outdoor fitness areas. Those existing works were effort-consuming, and some required excess GPS terminal devices, which is not desirable with a limited budget. As a result, they could not estimate the GPS positioning performance at the city scale.

## 3. Basic Concepts

**Definition** **1.** ***Road network.** The road network is a graph RN=(Nodes,Edges) comprised of a set of roads connected to each other in a graph format. $Edges=\{edge_{i}\}$ is the set of the edges with each edge associated with a road. $Nodes=\{node_{i}\}$ is the set of the nodes with each node associated with an intersection represented by $\left(id_{i},longitude_{i},latitude_{i}\right)$. Edge set Edges is a subset of the cross product N×N, where N is the number of nodes. Each element $edge(node_{i},node_{j})$ in Edges is a street connecting $node_{i}$ to $node_{j}$. In this work, the road is depicted as a line without any width. The road network data of Chengdu was downloaded from OpenStreetMap (Please check the official site of OpenStreetMap for more details: http://www.openstreetmap.org/).*

**Definition** **2.** ***Road segment.** A road segment $road_{i}$ of the road $edge_{j}$ is a continuous part of $edge_{j}$. A road could be divided into several road segments. In this paper, we set the length of a road segment equal to 50 m. The road whose length was less than 50 m was treated as a single road segment.*

**Figure 1:**
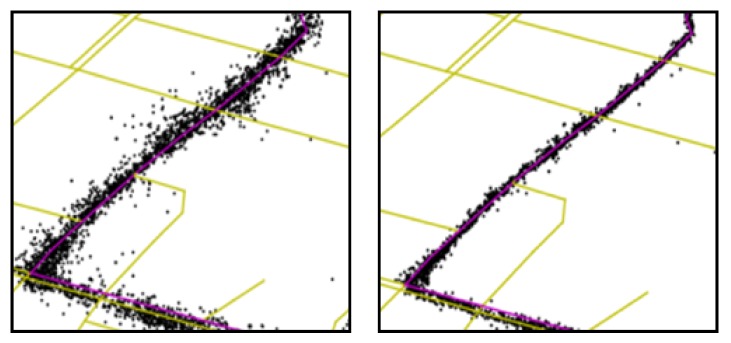
GPS trajectory data of two buses on the same roads.

**Definition** **3.** ***Bus route.** The bus route $BR_{i}$ is a subgraph of the road network graph RN. In this paper, there were 184 different bus lines in Chengdu that covered n=8831 road segments in road network RN. There was always more than one bus running on the same route. For example, the red lines in Figure 1 denote a part of the bus line route.*

**Definition** **4.** ***Bus trajectory.** The trajectory $G_{i}=\left\{g_{i,t}\right\}\left(i=1,\cdots ,m\right)$ of $bus_{i}$ is a sequence of GPS points $g_{i,t}$. We used m to denote the number of buses. m equalled 4835 in our work. The GPS point $g_{i,t}=\left(time_{i,t},latitude_{i,t},longitude_{i,t}\right)$ consists of a time-stamp $time_{i,t}$, a latitude record $latitude_{i,t}$, and a longitude record $longitude_{i,t}$. For example, the black points in Figure 1 denote the GPS trajectory data of buses.*

**Definition** **5.** ***POI information of the road segment.** The POI information of the road segment is depicted by several different POI categories from the online map. For $road_{i}$, we constructed a POI feature vector $c_{i}=\left(cnt_{1},\cdots ,cnt_{num}\right)$, where num denotes the number of different POI categories and $cnt_{j}\left(j=1,\cdots ,num\right)$ denotes the number of nearby (within 200 m) POI, which belong to category $poi_{j}$. Concretely in this paper, there were num=17 different POI categories according to the Gaode Online Map (Please check the official site of Gaode Map for more details: http://ditu.amap.com/): catering services, traffic infrastructures, government agency, vehicle sales, corporations, scenic spots, sports services, science education services, shopping services, accommodation services, vehicles services, serviced apartment, finance insurance services, life services, vehicle maintenance, and medical care services.*

**Definition** **6.** ***Tags of the road segment.** According to the OpenStreetMap, road segments could be categorized by tags: PrimaryLink, LivingStreet, service, residential, SecondaryLink, primary, MotorwayLink, unclassified, motorway, trunk, TrunkLink, tertiary, secondary (Please check the wiki of OpenStreetMap for more details of the tags: http://wiki.openstreetmap.org/wiki/Highway_link/). Each road segment is labelled with only one tag.*

**Definition** **7.** ***Layout information of the road segment.** The layout information of the road segment is depicted by several different floors. For $road_{i}$, we constructed a layout feature vector $h_{i}=\left(height_{1},\cdots ,height_{num}\right)$, where num denotes the number of different floors and $height_{j}\left(j=1,\cdots ,num\right)$ denotes the number of nearby (within 200 m) buildings with j floors. Concretely in this paper, there were num=60 different floors within the second-ring road in Chengdu, China.*

**Definition** **8.** ***GPS positioning bias.** The GPS positioning bias refers to the linear distance between the GPS positioning record and the real position of the bus. It ranges from a few meters in open sky environments to over 80m in urban canyons [7]. The positioning bias of a bus on the road could be divided into two orthonormal parts. One is vertical to the road, while the other is parallel with the road. The vertical component is much greater than the parallel component, which can be ignored [7,32]. In this paper, such bias is measured as the vertical distance between the GPS positioning point and the real road where the bus is running.*

**Figure 2:**
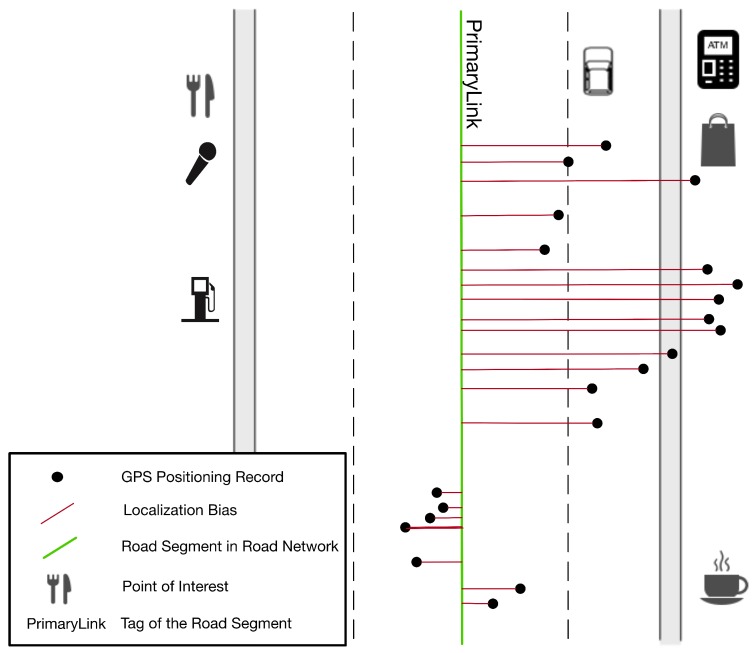
Meta information of the data.

**Definition** **9.** ***GPS positioning error.** The real horizontal position of the bus along the roads can be figured out based on map-matching algorithms. However, the width of the actual road cannot be ignored with regard to the GPS positioning bias. It is difficult to tell on which lane the bus is running. As a result, we utilized the standard deviation (std) of the GPS positioning biases to measure the buses’ GPS positioning errors on roads, instead of the mean values of the biases. In this way, the GPS positioning error is defined as the standard deviation (std) of the GPS positioning biases. Such error is affected by satellite ephemeris error, receiver clock error, multipath error, spherical error, receiver measurement noise, and so on. Multipath error is the major component when locating in urban areas. The concepts above are shown in Figure 2.*

**Definition** **10.** ***GPS environment friendliness (GEF).** Multipath error is caused by the delay of the signal arrival due to its reflection off building surfaces in the area. GPS environment friendliness defines the degree to which the multipath phenomenon affects the GPS performance. The GEF depends on the surrounding environment. It is independent of time, weather, the quality of GPS positioning terminal device, and the number of visible GPS satellites. We assumed that different locations within the same road segment shared a similar environment and the same GEF.*

**Figure 3:**
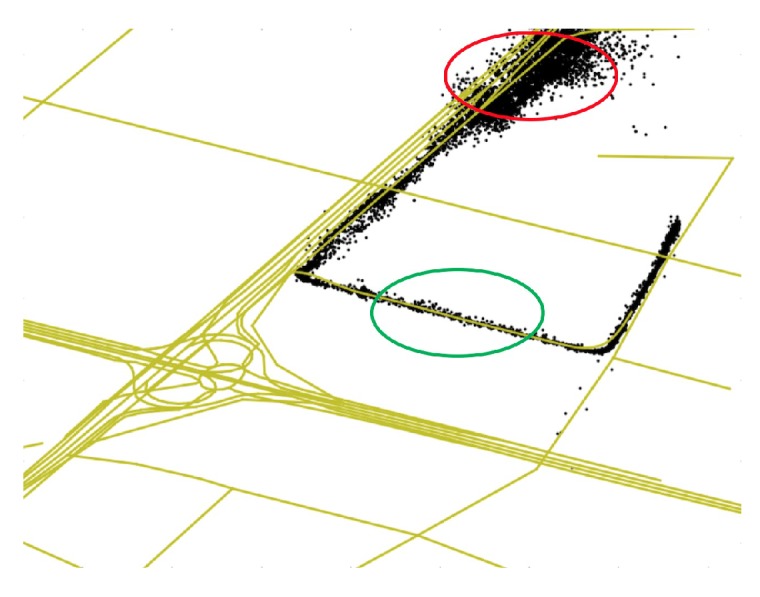
GPS trajectory data of one bus on different roads.

For a specific road segment, the GEF is considered poor if the std is high, while lower std indicates that the GEF is better. To understand the GEF introduced in this paper intuitively, we show the GPS trajectory data of one bus on different roads in Figure 3. Yellow lines denote the road network, and black points denote the GPS records of the bus. The GPS points in the green circle are densely distributed, which means that their variance is small. It is indicated that the std of the GPS positioning error of the bus on this road is small and the GEF here is good. On the contrary, the GEF of the road marked by the red circle is poor.

## 4. Methodology

### 4.1. Overview of the Framework

We developed an urban road GPS environment friendliness estimation approach based on the historical bus GPS trajectory data. The whole framework of the GEF evaluation was composed of the following main components:

1. We utilized the hidden Markov model (HMM)-based map matching algorithm [33,34,35,36] to map the bus trajectories’ data to the roads. The accuracy and efficiency of the map matching process were improved significantly based on the pre-knowledge of bus routes. After the map matching, we constructed a matrix, where the element of the matrix represented the positioning error standard deviation of each bus on each road segment. Note that the route of one bus only covered a small portion of the roads in the city. There were few buses running on any given road. Thus, the matrix to be completed was very sparse.
2. We estimated the positioning errors of each bus on each road segment based on the matrix completion algorithm, taking the nearby environment information into consideration. Due to the variance of the quality of the GPS receivers, an incorrect conclusion would be drawn if we estimated the GEF of a road only depending on the buses whose routes covered the road. Ideally, the GEF of a road is supposed to be estimated according to the GPS errors of all buses. Therefore, we needed to complete the matrix that was constructed in the first phase.
3. The GEF of each road segment was estimated based on the completion result. The buses whose GPS terminal device had a higher quality would have more weight on the evaluation of the GEF.

The details will be presented in the following subsections.

### 4.2. Map Matching-based GPS Error Matrix Construction

The observed GPS positions needed to be aligned with the road network on the digital map to conduct further analysis. This process is called map matching, which is a fundamental pre-processing step for trajectory-based research and applications [28,29,37,38]. We applied the HMM-based map matching algorithm as an algorithm prototype [33], which is based on two rules:

- As mentioned in Definition 8 and [7,32], the probability that a GPS point is matched to a road segment is related to the vertical distance between them. The shorter the distance is, the greater the probability is.
- Since the bus is continuously running on the road, the road segment corresponding to the current GPS sampling point should be close to the road segment corresponding to the previous point.

Based on the two above rules, the algorithm could calculate the emission probabilities and the transition probabilities and then use a dynamic planning strategy (Viterbi algorithm) to find the best matched path.

However, if we applied the above algorithm prototype to our bus trajectory dataset directly, the amount of calculation would be relatively large. It is difficult for the map matching algorithm to achieve good performance when applied directly to trajectories with large errors [28]. In fact, compared to the trajectory data of other vehicles (such as taxi data), bus trajectory data have their own characteristics. Fixed bus routes data can provide important supplementary information for map matching, which can reduce the number of candidate roads and improve the computational efficiency and matching accuracy. Concretely, a bus usually ran on its specific route. Therefore, the real position of each GPS point was believed to be located on the nearest road segment that was covered by the route of this bus, and the GPS positioning error of the record could be calculated. Besides, there were several buses running on the same fixed line, and each of them went through the routes many times under different weather conditions and in time periods. As a result, the error produced by map matching and accidental factors (e.g., the position of satellites, weather) was reduced to some extent.

Concretely, we divided roads in the road network into short equal-length road segments. On the one hand, we assumed that the GEF at different locations within the same road segment was the same. On the other hand, the GEF of a segment could be estimated only if there were enough GPS record points.

**Figure 4:**
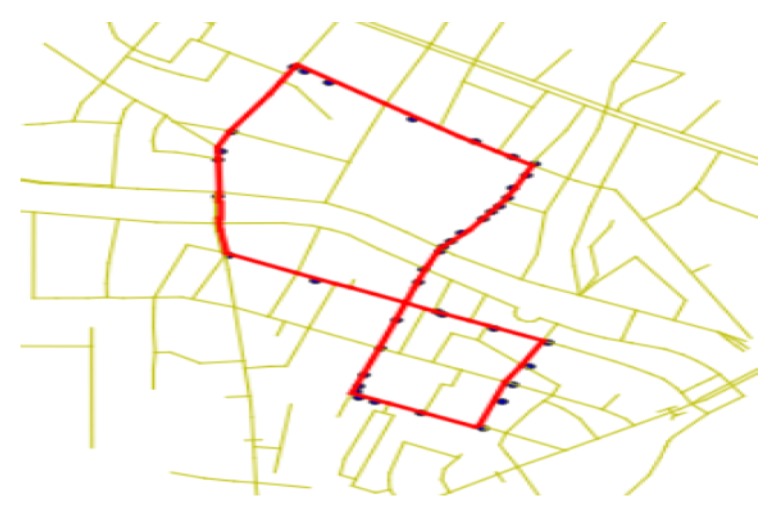
Map matching result of Bus Line 1022 route data.

As the bus route data provided by the public transport company were also designated by GPS points, we then needed to match bus route data to the road network. After that, the bus routes were designated by road segments, to which bus trajectory data would be mapped (i.e., the red lines in Figure 4). Figure 4 shows the map matching result of bus route data of Line 1022. The yellow lines denote roads in Chengdu. The black points denote the GPS point of line 1022 route data. The red lines denote the map matching results of the black points.

Some GPS record points could not be mapped to the bus routes within the threshold distance. The main reason for the failure of map matching was that the bus did not travel exactly on the given route. Bus route data provided by the bus company may not be entirely accurate because of a temporary road diversion for construction or the delayed update of bus route data after route adjustment. The bus may not travel on a given route because of repair or refuelling. What is more, the bus may be temporarily scheduled to travel on another route. If there were more than five consecutive points far away (more than 50 m) from the given route, the bus was believed to be veering off its route. Those points would be mapped to other nearby segments on the road network until the bus returned back to its given routes.

### 4.3. GPS Error Estimation with Additional Environment Information Integration

After mapping each GPS record to the corresponding road segment, we calculated the positioning biases of each bus on every passing road segment. The standard deviation of biases was utilized to measure the error. Matrix **Var** was then constructed, where the entry $v_{ij}$ denotes the error of $bus_{i}$ on $road_{j}$. However, there existed no bus that could pass all roads, making this matrix very sparse.

The routes of all buses in total had a high coverage for roads in the city, while the bus trajectory data of a single bus was quite sparse in the city. For example, within the second-ring of Chengdu (the city we focused on in this study), a single bus’ coverage was only about 2.5%. The GEF of roads in the city had to be estimated based on the GPS records of many buses instead of a single bus.

However, there was a significant variance in the quality of GPS receivers among different buses, which meant that the GPS positioning performance of different buses on the same road segment may differ from each other. Therefore, the GPS samples of different buses could not be merely aggregated to solve the low-coverage problem of a single bus. Therefore, a high GPS error may be caused by GPS receivers with low quality, even on the road segment with good GEF. To reduce such negative influence and estimate the GEF of road segments fairly, we needed to estimate the error of each bus on each road segment. In order for the influence brought by the variance of GPS receivers’ quality to be eliminated, we could compare the GEF on different road segments more fairly.

To complete a matrix, compressive sensing is widely applied [39]. Given a sparse matrix for which most of its items are missing, compressive-based matrix completion will estimate those missing items according to the specific cost function and optimization algorithm. In addition to the common cost function, we tried to incorporate prior knowledge in our completion framework, i.e., the nearby building layout information of roads. With the above prior knowledge, it was possible for us to estimate missing items in the matrix more precisely.

#### 4.3.1. Basic Objective Function of Matrix Completion

After the map matching process, we constructed a matrix **Var** recording the standard deviation of GPS positioning biases, which measured the errors on road segments:

$$\begin{array}{c} \mathrm{Var}=\left(\begin{array}{ccc} v_{11} & \cdots & v_{1n}\\ \vdots & \ddots & \vdots \\ v_{m1} & \cdots & v_{mn} \end{array}\right)={\left(\begin{array}{c} \vec{v_{1}}\\ \vdots \\ \vec{v_{m}} \end{array}\right)}_{m\times n} \end{array}$$

$v_{ij}$ denotes the bias std of $bus_{i}$ on road segments $road_{j}$. Row vector $\vec{v_{i}},\;\left(i=1,\cdots ,m\right)$ denotes the errors of $bus_{i}$ on each road segment. Column vectors denote errors of each bus on the given road segment. If the number of GPS records of $bus_{i}$ on $road_{j}$ was less than 20, the $v_{ij}$ would be set as a missing value. Note that the matrix **Var** was to be completed and could be very sparse. The basic objective function of matrix completion was set as [39]:

(1) $$\begin{array}{c} F\left(\mathrm{Sign},\mathrm{Var},L,R\right)=||\mathrm{Sign}\cdot {\mathrm{LR}}^{T}-{\mathrm{Var}||}_{F}^{2}+{\lambda (||L||}_{F}^{2}+{\left||R|\right|}_{F}^{2}) \end{array}$$

The size of binary identification matrix **Sign** was the same as matrix **Var**. $s_{ij}$ equalled 1 if $v_{ij}$ was known. Otherwise, $s_{ij}$ equalled 0. $s_{ij}=1_{\left\{\left(i,j\right)|v_{ij}\;is\;known.\right\}}$. The result of matrix completion was ${\mathrm{LR}}^{T}$. The size of matrix **L** was m×a, and the size of matrix **R** was n×a. *a* was a hyper-parameter of matrix completion. The penalty term $||\mathrm{Sign}\cdot {\mathrm{LR}}^{T}-\mathrm{Var}{\left|\right|}_{F}^{2}$ measured the similarity between the completion result and original matrix. ${\left||L|\right|}_{F}^{2}+{\left||R|\right|}_{F}^{2}$ was the regularization term. λ was the hyper-parameter denoting the importance of the penalty term.

#### 4.3.2. Measure the Relative Advantage of GPS Receivers’ Qualities

To measures the relative advantage of GPS positioning terminals’ qualities between each of two buses, an m×m matrix **Qua** was constructed. To test the equality of variations, we used the F-test [40], initially developed by A.Fisher. The hypothesis was that the means of a given set of normally distributed populations, all having the same standard deviation, were equal. Under the Gaussian assumption, any scaled pair of variations of our sample could form a pivot variable following an F distribution if the null hypothesis was true. Then, we could perform hypothesis tests on any pair of variations at the level of 5%.

$$\begin{array}{c} \mathrm{Qua}={\left(\begin{array}{ccc} k_{11} & \cdots & k_{1m}\\ \vdots & \ddots & \vdots \\ k_{m1} & \cdots & k_{mm} \end{array}\right)}_{m\times m} \end{array}$$

where $k_{ij}$ measures the relative advantage of $bus_{i}$ over $bus_{j}$.

$$\begin{array}{c} k_{ij}=\left\{\begin{array}{cc} 1 & \mathrm{if}\;\mathrm{the}\;\mathrm{quality}\;\mathrm{of}\;bus_{i}\;\mathrm{is}\;\mathrm{better}\;\mathrm{than}\;bus_{j},\\ -1 & \mathrm{if}\;\mathrm{the}\;\mathrm{quality}\;\mathrm{of}\;bus_{i}\;\mathrm{is}\;\mathrm{worse}\;\mathrm{than}\;bus_{j},\\ 0 & \mathrm{if}\;\mathrm{the}\;\mathrm{relative}\;\mathrm{advantage}\;\mathrm{cannot}\;\mathrm{be}\;\mathrm{determined},\;\mathrm{or}\;i=j. \end{array}\right. \end{array}$$

Concretely, $bus_{i}$ and $bus_{j}$ only compared with each other on $road_{r}$, which has the most GPS points of them. It was assumed that GPS errors followed a Gaussian distribution. Thus, we performed an F-test between the GPS error sequences of $bus_{i}$ and $bus_{j}$ on $road_{r}$, while the confidence coefficient was 95%. As a result, the quality of $bus_{i}$ was considered as better than the quality of $bus_{j}$, if $v_{ir}<v_{jr}$.

However, if there was not any road that had been travelled by both $bus_{i}$ and $bus_{j}$, we would try to find another intermediate $bus_{q}$. The quality of $bus_{i}$ was considered as better than the quality of $bus_{j}$, if $k_{iq}=1$ and $k_{qr}=1$, while both of the confidence coefficients should be higher than 97.5%; or the relative advantage between $bus_{i}$ and $bus_{j}$ was considered not able to be determined. In order to make the matrix completion result meet the relative advantage between different buses, we constructed an m×m matrix **Tran** based on matrix **Qua**.

$$\mathrm{Tran}={\left(\begin{array}{cccc} \Sigma_{j=1}^{m}k_{1j} & -k_{12} & \cdots & -k_{1m}\\ -k_{21} & \Sigma_{j=1}^{m}k_{2j} & \cdots & -k_{2m}\\ \vdots & \vdots & \ddots & \vdots \\ -k_{m1} & -k_{m1} & \cdots & \Sigma_{j=1}^{m}k_{mj} \end{array}\right)}_{m\times m}$$

Consider the transformation of matrix **Var**:

$$\begin{array}{cc} \mathrm{Tran}\cdot \mathrm{Var} & =\left(\begin{array}{ccc} \Sigma_{j=1}^{m}k_{1j} & \cdots & k_{1n}\\ \vdots & \ddots & \vdots \\ k_{m1} & \cdots & \Sigma_{j=1}^{m}k_{mj} \end{array}\right)\left(\begin{array}{c} \vec{v_{1}}\\ \vdots \\ \vec{v_{m}} \end{array}\right)\\ & ={\left(\begin{array}{c} \Sigma_{j=1}^{m}k_{1j}\vec{v_{1}}-k_{12}\vec{v_{2}}-\cdots -k_{1m}\vec{v_{m}}\\ \vdots \\ -k_{m1}\vec{v_{1}}-k_{m2}\vec{v_{2}}-\cdots -+\Sigma_{j=1}^{m}k_{mj}\vec{v_{m}} \end{array}\right)}_{m\times n} \end{array}$$

To get better insight into this transformation, consider $Row_{1}$ of Tran·Var.

$$\begin{array}{c} Row_{1}\left(\mathrm{Tran}\cdot \;\mathrm{Var}\right)\\ \;=\Sigma_{j=1}^{m}k_{1j}\vec{v_{1}}-k_{12}\vec{v_{2}}-\cdots -k_{1m}\vec{v_{m}}\\ \;=\Sigma_{j=2}^{m}k_{1j}\vec{v_{1}}-k_{12}\vec{v_{2}}-\cdots -k_{1m}\vec{v_{m}}\\ \;=\Sigma_{j=2}^{m}k_{1j}\cdot \left(\vec{v_{1}}-\vec{v_{j}}\right)\\ \;=\Sigma_{j=2}^{m}k_{1j}\cdot \left(v_{11}-v_{j1},\;v_{12}-v_{j2},\;\cdots ,\;v_{1m}-v_{jm}\right)\\ \;=(\;\Sigma_{j=2}^{m}k_{1j}\left(v_{11}-v_{j1}\right),\;\cdots ,\;\Sigma_{j=2}^{m}k_{1j}\left(v_{1m}-v_{jm}\right)\;{)}_{1\times m} \end{array}$$

Recall the construction of $k_{ij}$; ideally, the value of $k_{ij}\left(v_{il}-v_{jl}\right),l=1,\cdots ,m$ should be a negative value for all (i,j) pairs. Thus, every input of Tran·Var should be a negative value in the ideal case, while a positive value is an inappropriate input.

Due to the lack of a zero lower bound of the Tran·Var F-norm, the cost function would not converge if we added this matrix into the cost function directly. Consider a monotone matrix operation $E_{\theta }\left(\cdot \right),\theta >0$: θ is a predetermined positive number controlling the absolute values of $e^{\theta y}$s to avoid overflow while processing the algorithm. Here, $\theta =max{\left(y_{ij}\right)}^{-1},\;\left(i=1,2,\cdots ,m;\;j=1,2,\cdots ,n\right)$.

$$E_{\theta }\left(Y\right)=\left(\begin{array}{ccc} e^{\theta y_{11}} & \cdots & e^{\theta y_{1n}}\\ \vdots & \ddots & \vdots \\ e^{\theta y_{m1}} & \cdots & e^{\theta y_{mn}} \end{array}\right)$$

where **Y** is an arbitrary m×n matrix:

$$\begin{array}{c} Y=\left(\begin{array}{ccc} y_{11} & \cdots & y_{1n}\\ \vdots & \ddots & \vdots \\ y_{m1} & \cdots & y_{mn} \end{array}\right) \end{array}$$

The preferred properties of matrix operation $E_{\theta }\left(\cdot \right)$:

1. The elements in $E_{\theta }\left(\mathrm{Tran}\cdot \mathrm{Var}\right)$: inherit the relative magnitudes of the elements in Tran·Var, small values for the ideal case, large values for an inappropriate case.
2. It guarantees a lower bound of $\left|\right|E_{\theta }\left(\mathrm{Tran}\cdot {\mathrm{LR}}^{T}\right){\left|\right|}_{F}^{2}$, so that the objective function below has a lower bound. Thus, it is possible to converge when we solve the system iteratively.

As a result, we added the penalty below to the objective function:

$$\begin{array}{c} \lambda_{2}\left(|\right|E_{\theta }\left(\mathrm{Tran}\cdot {\mathrm{LR}}^{T}\right){\left|\right|}_{F}^{2}) \end{array}$$

#### 4.3.3. Measure the POI Information of Road Segments

The GEF of road segments with tall buildings would be more likely to be poor due to the urban canyon phenomenon; while it may good if there is an open square near a road segment. We believed that the POI information of road segments was able to characterize the nearby building layout environment. For example, there may be more POI of catering services and shopping services on the road segments with tall buildings. We assumed that the GEF of two roads was similar to each other, when the Euclidean distance between two POI vectors annotating two roads was small. According to Gaode Map, the road was depicted by 17 different POI categories. For $road_{i}$, we constructed a POI feature vector $\vec{c_{i}}$:

$$\vec{c_{i}}=\left(\begin{array}{ccc} cnt_{1}\; & \cdots \; & cnt_{17} \end{array}\right)$$

where $cnt_{q}\left(q=1,\cdots ,17\right)$ is the number of nearby (within 200 m) POI, which belong to $category_{q}$. Then, compute the Euclidean distance between each POI vector of roads segments:

$$\begin{array}{c} \mathrm{Dist}=\left(\begin{array}{ccc} d_{11} & \cdots & d_{1n}\\ \vdots & \ddots & \vdots \\ d_{m1} & \cdots & d_{mn} \end{array}\right) \end{array}$$

where $d_{ij}$ denotes the Euclidean distance between the POI vector of $c_{i}$ and $c_{j}$. Thus, we can construct matrix **Poi** to describe the similarity of the POI distribution between each of two roads.

$$\mathrm{Poi}=\left(\begin{array}{ccc} p_{11} & \cdots & p_{1n}\\ \vdots & \ddots & \vdots \\ p_{n1} & \cdots & p_{nn} \end{array}\right)$$

where:

$$\begin{array}{c} p_{ij}=\left\{\begin{array}{cc} 0 & EuclideanDistance(r_{i},r_{j})>\varepsilon ,\\ \frac{1/d_{ij}}{\Sigma_{k}1/d_{ik}} & EuclideanDistance(r_{i},r_{j})<\varepsilon . \end{array}\right. \end{array}$$

*k* denotes the number of road segments, which had a similar POI distribution as $road_{i}$. In the Experiment Section, ε was set to 250, tuned by 3-fold cross-validation. According to our assumption, the objective function should be penalized if there was a big difference between GPS errors of buses on roads, whose POI distributions were similar to each other. As a result, we added the penalty below to the objective function:

$$\begin{array}{c} \lambda_{3}\cdot \Sigma_{i=1}^{n}\overset{n}{\underset{j=1,j\neq i}{\Sigma }}\frac{1}{d_{ij}}\left|\right|{\mathrm{LR}}^{T}p_{ij}{\left|\right|}_{F}^{2} \end{array}$$

#### 4.3.4. Measure the Tag Information of Road Segments

According to the OpenStreetMap, road segments could be categorized by tags (e.g., PrimaryLink, LivingStreet). Similar to the POI distribution, it was also assumed that the GEF of roads would be similar, if they had the same tag. As a result, we constructed the matrix **Tag** $={\left(t_{ij}\right)}_{n\times n}$.

$$\mathrm{Tag}=\left(\begin{array}{ccc} t_{11} & \cdots & t_{1n}\\ \vdots & \ddots & \vdots \\ t_{n1} & \cdots & t_{nn} \end{array}\right)$$

$$\begin{array}{c} t_{ij}=\left\{\begin{array}{cc} -1 & i=j,\\ 1/(k-1) & \mathrm{if}\;road_{i}\;\mathrm{and}\;road_{j}\;\mathrm{have}\;\mathrm{the}\;\mathrm{same}\;\mathrm{tag},\\ 0 & \mathrm{otherwise}. \end{array}\right. \end{array}$$

*k* denotes the number of road segments that have the same tag as $road_{i}$ and $road_{j}$. According to our assumption, the objective function should be penalized if there was a big difference between the GPS errors of buses on roads, which belonged to the same tag category. The new regularization term was designed as:

$$\begin{array}{c} \lambda_{4}\cdot \left|\right|{\mathrm{LR}}^{T}\mathrm{Tag}{\left|\right|}_{F}^{2} \end{array}$$

#### 4.3.5. Measure the Layout Information around Road Segments

The GEF of road segments with tall buildings around would be more likely to be poor due to the urban canyon phenomenon, while it may be good if there was an open square near the road. It was assumed that the layout information around road segments was able to characterize the nearby environment. The GEF of two roads should be similar to each other if the nearby building environments were similar as well. We assumed that the GEF of two roads was similar to each other, when the Euclidean distance between two layout vectors annotating two roads was small. For example, there may be more urban canyons or other terrain that lead to poor GEF on the road segments with tall buildings.

The number of floor levels in Chengdu ranged from 1 to 60. Therefore, the layout of each road segment was depicted as a 60-dimensional vector, which meant the number of buildings (within 200 m) of each corresponding height. For $road_{i}$, we constructed a layout feature vector $\vec{h_{i}}$ to depict its nearby building layout:

$$\vec{h_{i}}=\left(\begin{array}{ccc} height_{1} & \cdots & height_{60} \end{array}\right)$$

where $height_{q}\left(q=1,\cdots ,60\right)$ is the number of nearby (within 200 m) buildings that belongs to *q* floors. Then, we computed the Euclidean distance between each height vector of road segments: **Dist** $={\left(d_{ij}\right)}_{m\times n}$. $d_{ij}$ denotes the Euclidean distance between the layout vector of $h_{i}$ and $h_{j}$. Thus, we could construct matrix **Layout** $={\left(l_{ij}\right)}_{n\times n}$ to describe the similarity of the layout between each of two segments.

$$\begin{array}{c} l_{ij}=\left\{\begin{array}{cc} 0 & EuclideanDistance(h_{i},h_{j})>\varepsilon ,\\ \frac{1/d_{ij}}{\Sigma_{k}1/d_{ik}} & EuclideanDistance(h_{i},h_{j})<\varepsilon . \end{array}\right. \end{array}$$

*k* denotes the number of road segments that have a similar layout as $road_{i}$. According to our assumption, the objective function should be penalized if there was a big difference between the GPS errors of buses on roads, whose layouts were similar to each other. As a result, we added the penalty to the objective function, and the final objective function of matrix completion was:

(2) $$\begin{array}{c} \begin{array}{c} F\left(\mathrm{Sign},\mathrm{Var},L,R,\mathrm{Tran},\mathrm{Poi},\mathrm{Tag}\right)=||\mathrm{Sign}\cdot {\mathrm{LR}}^{T}-{\mathrm{Var}||}_{F}^{2}+\lambda_{1}{\left(||L|\right|}_{F}^{2}+{\left||R|\right|}_{F}^{2})+\lambda_{2}\left(|\right|E_{\theta }\left(\mathrm{Tran}\cdot {\mathrm{LR}}^{T}\right){\left|\right|}_{F}^{2})\\ +\lambda_{3}\Sigma_{i=1}^{n}\overset{n}{\underset{j=1,j\neq i}{\Sigma }}\frac{1}{d_{ij}}\left|\right|{\mathrm{LR}}^{T}p_{ij}{\left|\right|}_{F}^{2}+\lambda_{4}\left|\right|{\mathrm{LR}}^{T}{\mathrm{Tag}||}_{F}^{2}+\lambda_{5}\Sigma_{i=1}^{n}\overset{n}{\underset{j=1,j\neq i}{\Sigma }}\frac{1}{d_{ij}}\left|\right|{\mathrm{LR}}^{T}l_{ij}{\left|\right|}_{F}^{2} \end{array} \end{array}$$

#### 4.3.6. Optimization of the Objective Function

The general objective function of matrix completion can be solved iteratively, where each iteration consists of two steps [39]. First, for a given fixed L, update R element-wisely in the gradient descent direction of the objective function. Second, fixing the updated R, update L element-wisely in the same manner.

However, it was intractable to adopt the gradient descent method because of the term $\lambda_{2}\left(|\right|E_{\theta }\left(\mathrm{Tran}\cdot {\mathrm{LR}}^{T}\right){\left|\right|}_{F}^{2})$ in our objective function. The computational complexity in a single iteration to update terms was $O(m^{2}n^{2})$, and m×n was the size of the input matrix. If we took 50 m as the length of the road segment, then the complexity was about $O(4835^{2}\times 8831^{2})$, which was intolerably high.

Here, we took advantage of the simulated annealing algorithm to get a more effective solution. An empirically well-adopted initialization [39,41] of L and R is given by non-negative matrix factorization (NMF) [42]. The detail pseudocode of algorithm is shown in Algorithm 1.
**Algorithm 1:** Matrix completion.
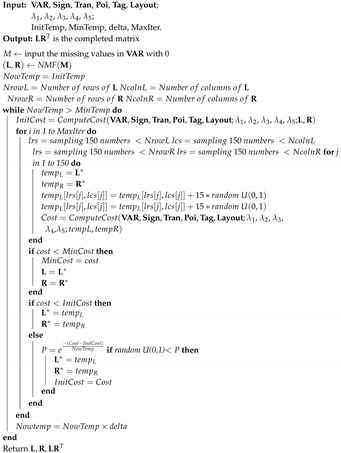


### 4.4. Weighted Estimation of GEF

After completing the GPS positioning error matrix, we obtained the approximate GPS positioning error for each bus on each road segment. Our goal was to rank the road segments based on GPS environment friendliness. However, considering the different quality of GPS terminal devices on different buses, we needed to give buses different weights when estimating the GEF of road segments. The intuition was that the bus with a high-quality GPS terminal device could better distinguish between road segments. The quality of the GPS receivers of most buses was acceptable. The GPS receiver would be considered as unconvincing if its positioning performance was significantly different from other buses on the same road segment. We used $distinction_{i}$ and $consistency_{i}$ to measure the weight of $bus_{i}$. $distinction_{i}$ represents the capacity of $bus_{i}$ to distinguish between road segments. $consistency_{i}$ represents the degree of $bus_{i}$’s consistency with other buses on the same road segment.

$$distinction_{i}=std\left(v_{i,:}\right)$$

$$consistency_{i}=\frac{1}{mean({\left[\frac{v_{ij}-mean\left(v_{:,j}\right)}{std(v_{:,j})}\right]}_{j=1,2,\cdots ,n})}$$

$$weight_{i}=distinction_{i}\cdot consistency_{i}$$

Given the weights of buses, we could calculate the average error of $road_{j}$ as follows:

$$\begin{array}{c} GEF_{j}=\frac{\Sigma_{i=1}^{m}\left(weight_{i}\cdot v_{ij}\right)}{\Sigma_{k=1}^{m}weight_{k}} \end{array}$$

After that, we ranked road segments based on average errors. The smaller the average error was, the better the GEF was.

## 5. Experiment

In this section, we estimate the GEF of roads covered by bus routes within the second-ring road in Chengdu, China. The estimation results were compared with the baseline methods. We also selected several road segments to collect real-life GPS measurements as the ground-truth to verify the rationality of the results by a case study.

### 5.1. Dataset Description

The data we used were from a real-world dataset collected in Chengdu, China. The GPS points were recorded by the buses running on their fixed routes for 30 days (2015.11.01–2015.11.30), which meant that the GPS readings were recorded under different conditions (e.g., different weather conditions and satellite positions). For each bus, it generated 2 to 4 records per minute, and thus, the total number of GPS point records was about 62,783,000, which was far more than the existing field-test works. The basic statistics about the data are shown in Table 1.

The urban road network was obtained from OpenStreetMap (Please check the official site of OpenStreetMap for more details: http://www.openstreetmap.org/). The urban road network was divided into short and equal-length road segments, so that the GEF at different locations within the same segment could be treated as the same.

As mentioned above, there was a significant variance in the quality of GPS receivers among different buses. This was because buses were managed by different public transportation operating companies, and the time of GPS installation and update varied from each other, which lead to the diversity in GPS receivers’ brands and models. Taking the city of Chengdu as an example, there were more than 80 different types of GPS receivers in 4835 buses. For different GPS receivers, the quality varied obviously. To understand the difference intuitively, we show the GPS trajectory data of two buses on the same roads in Figure 1. Yellow lines denote the road network. Black points denote the GPS records of the buses. Red lines denote the route where the buses are running. Obviously, the GPS positioning accuracy of the first bus was worse than the second bus.

### 5.2. Result of Map-Matching

About 80.90% (50,789,815) of the GPS points were mapped to their given bus routes under the distance threshold. About 81.09% of such remaining (19.10%) points were mapped to nearby road segments under the distance threshold. As a result, 96.39% (60,517,304) of all points were mapped successfully. Other points were abandoned as accidental outliers.

### 5.3. Evaluation of the Matrix Completion Result

We employed *k*-fold cross-validation to evaluate the precision of the completed results of our completion algorithm. Concretely, *k* was set as 3 in this experiment. We used estimate error to measure the accuracy of matrix completion.

In detail, all non-zero positions of matrix **Var** were equally divided into *k* parts $\left(P_{1},P_{2},\cdots ,P_{K}\right)$. For each part $P_{i}$, we covered it and preserved the remaining k−1 parts. We applied our completion algorithm to matrix Var and obtained the completed matrix ${\mathrm{LR}}^{T}$. We calculated the estimate-error [43] according to ${\mathrm{LR}}^{T}$ as follows: $\xi_{i}=\frac{\sum_{r,t:v_{r,t}\in P_{i}}\left|v_{r,t}-{\mathrm{LR}}_{r,t}^{T}\right|}{\sum_{r,t:v_{r,t}\in P_{i}}\left|v_{r,t}\right|}$. Enumerate the covered part from $P_{1}$ to $P_{k}$, and calculate the final estimate-error as: $\xi =\frac{\sum_{i=1}^{k}\xi_{i}}{k}$. Repeat the above operations *t* times, and calculate the average estimate error as the evaluation result of the completion algorithm. The rank comparing result is shown in Table 2, and we can see that our method outperformed the following baseline methods:

**Naive KNN**: For each empty entry in one row (column), we searched the *k* nearest rows (columns) whose corresponding entry was not null according to the Euclidean distance. Then, KNN used these non-empty entries to do the estimation.

**Correlation-based KNN**: This was similar to naive KNN. The only difference was that it used the correlation to measure the similarity instead of the Euclidean distance.

**Non-negative matrix factorization (NMF)** [42]: The matrix was factorized into two matrices, with the property that all matrices had no negative elements. Matrix multiplication of the factorized matrices was the completion result.

Our proposed algorithm consistently outperformed the baseline methods, which showed the superiority of our approach over other methods. The layout information, as well as the POI information represented the arrangement of the buildings at both sides along the road, and the tag information indicated the width of the road. They measured the signal occlusion effect to some extent. When integrating this prior information as additional penalty terms into the algorithm, the matrix completion performance was improved. Besides, it was also necessary to consider the variance between receivers’ qualities when estimating the error.

### 5.4. Case Study

It is extremely hard to evaluate the GPS positioning accuracy with ground-truth measurements in the whole city due to the cost. Case studies are common practices in related works [7,13,28]. We collected the ground-truth through a field study on six road segments and conducted the case studies to make an overall convincing comparison between our approach and the baseline methods. During all tests, all receiver outputs were obtained by an Android smartphone (HUAWEI GRA-CL00). A receiver moved along each road to generate GPS trajectories. About 200 GPS measurements were collected on each road segment.

**Figure 5:**
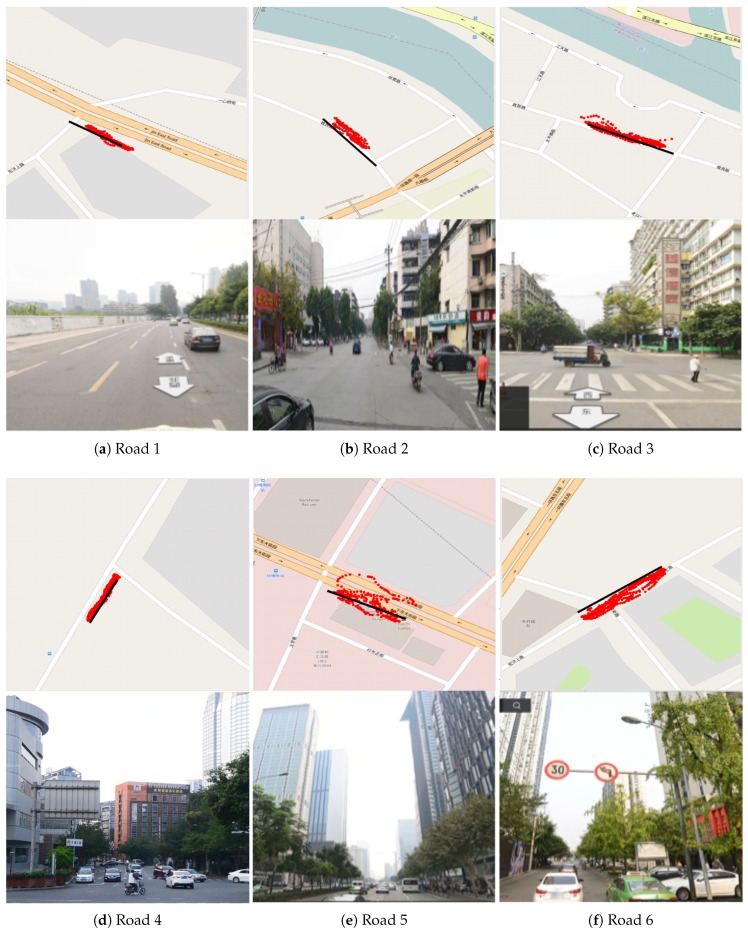
Field testing results and street views of road segments.

The results of the field tests are summarized in Table 3. The baseline methods directly took the average value of GPS measurements’ standard deviations of the buses that ran on the given road as the estimated GEF score. The GPS records and the street views of road segments are shown in Figure 5. The black line represents the real walking route marked manually on the map. Red points are the GPS positioning sequence records.

Our approach produced poorer GEF estimation for the 5th segment and satisfied estimation for the 2nd–4th segments. Both our approach and the baseline method produced similar estimation for the 1st segment (satisfied GEF) and 6th segment (poor GEF). According to the result of field tests, the GPS errors on Roads 1–4 were low, which meant that the GEF was good. The street views of Roads 1–4 also showed that the nearby buildings and trees were not so high, and the viewing range was wide, while the GPS errors on Roads 5–6 were high, which meant that the GEF here was poor. The street views of Roads 5–6 showed that there were many dense tall buildings on both sides of the roads. For Road Segments 1 and 6, both our approach and the baseline method gave the correct estimation. However, the baseline method degraded the GEF of Road Segments 2–4 and overrated the GEF of Road Segment 5. Field tests showed that our method estimated the GEF of these road segments correctly and outperformed the baseline method.

## 6. Limitation and Future Work

There were still a few limitations of this work.

1. Although the bus routes could cover most of the primary roads in the city, there were still plenty of bypasses whose GEF could not be estimated. However, our approach could be easily applied to trajectory data of taxis to tackle those bypasses, which is the future work. Besides, using the results of GEF assessment as the training data, environmental attributes could be extracted from urban street view pictures. Those attributes could be employed to estimate the GEF of cities without bus trajectory data.
2. There were only a few road segments where we conducted case studies due to the cost. Real-life GPS measurements on more road segments are expected to be collected, which is the future work.
3. We intend to apply our approach to location-based services and improve the user experience. Specifically, a model assessing the confidence level of real-time bus location and predicted arriving time could be modified from the GEF evaluation method.

## 7. Conclusions

We proposed a method for assessing the GPS environment friendliness of urban road segments based on processing and analysing massive historical bus GPS trajectory data. This method first took advantage of the unique feature that bus routes are fixed to construct the mapping from GPS data to road segments. Secondly, the missing data were completed based on the inherent correlation among GPS errors and the environment information. Finally, we put forward a weighted evaluation strategy to estimate the GEF, taking full consideration of the influence of the different GPS devices’ qualities. We exploited 4835 buses’ one-month trajectory data within the second-ring road of Chengdu to evaluate the GEF of 8831 different road segments, and the rationality of results was verified by satellite maps, street views, and field tests.

## Figures and Tables

**Table 1:** Dataset description.

Bus Line Number	184
Bus Number	4835
Duration	30 days
GPS Point Record Number	62,783,000
Sampling Rate of GPS Receiver	2–4 points/min
Number of Types of GPS Receivers	>80
Length of Road Segment	50 m
Road Segment Number	8831
Average of Buses Running on Each Segment	121
Average of Segments Covered by Each Bus Line	171
Number of GPS Points a Bus Recorded on a Segment	>20

**Table 2:** The estimate error of our method and baseline methods. NMF, negative matrix factorization.

Methods		Matrix Completion Error
	NAKNN	0.37242
Baseline Approaches	CBKNN	0.32951
	NMF	0.31883
	Basic Method (1)	0.29371
Our Approach	Integrating Layout Information	0.29348
	Integrating Layout and Tag and POI	0.29311
	Integrating All Penalty Terms (2)	**0.29220**

**Table 3:** Field tests results. GEF, GPS environment friendliness.

Road	Baseline		Our Approach		std of GPS Biases in Field Tests (m)
	Rank	GEF	Rank	GEF	
1	87	Satisfied	10	Satisfied	1.960
2	5307	Poor	1851	Satisfied	3.283
3	5312	Poor	2325	Satisfied	3.378
4	5177	Poor	326	Satisfied	1.492
5	759	Satisfied	6012	Poor	9.165
6	5085	Poor	5043	Poor	5.035
